# Repeated implantation failures and infertility in patients are strongly associated with elevated integrin B3 caused by endometrial copy number variation

**DOI:** 10.1530/RAF-24-0088

**Published:** 2025-03-21

**Authors:** Hong-Xia Xu, Sheng-Ni Liu, Xiao-Yi Xiang, Yan Lei, Yun-Xiu Li, Xiang-Jing Tang, Jian-Mei Yu, Li-Mei Tao, Ze Wu, Li Li

**Affiliations:** ^1^ Department of Reproductive Medicine, NHC Key Laboratory of Healthy Birth and Birth Defect Prevention in Western China, First People’s Hospital of Yunnan Province, Kunming, Yunnan, China; ^2^ The Affiliated Hospital of Kunming University of Science and Technology, Kunming, Yunnan, China; ^3^ University of Bristol, Faculty of Life Sciences, Bristol, UK; ^4^ Yunnan Medical Center for Pediatric Diseases, Yunnan Institute of Pediatrics, Kunming Children’s Hospital, Kunming Medical University, Kunming, Yunnan, China; ^5^ Kunming Key Laboratory of Children Infection and Immunity, Yunnan Key Laboratory of Children’s Major Disease Research, Yunnan Province Clinical Research Center for Children’s Health and Disease, Kunming, Yunnan, China; ^6^ Department of Gynecology, Hubei Province Women and Children Hospital, Wuhan, China

**Keywords:** recurrent implantation failure, gene copy number variations, integrin αvβ3, coagulation function, personalized reproductive medicine

## Abstract

This study reveals the association between gene copy number variants (CNVs) and integrin expression in endometrial tissue, especially focusing on patients with repeated implantation failure and unexplained infertility. CNV expression, integrin αVβ3 (by real-time quantitative polymerase chain reaction and protein immunoblotting), and related clinical indicators were investigated by analyzing endometrial samples from 48 patients with repeated implantation failure and ten patients with infertility who had no history of implantation issues. The results revealed the presence of CNVs in approximately 31.04% of patients with infertility, mainly affecting chromosomes 2, 5, 6, 7, 10, 11, 15, 19 and X. Age was associated with CNV occurrence. Integrin β3 expression was significantly higher in the CNV group than in the non-CNV group among patients with repeated implantation failure. Clinical indicators related to coagulation were significantly different between the CNV and non-CNV groups. The study indicated a potential association between CNVs, increased integrin β3 expression and recurrent implantation failure. The study provides new information for understanding the complex interactions between genetic variants, integrin function and coagulation factors by integrating advanced molecular diagnostic techniques, thereby emphasizing the need for a personalized approach in reproductive medicine. These results may redefine the diagnostic paradigm for recurrent implantation failure, laying the foundation for future translational applications.

**Lay summary:**

Our research has found a link between certain genetic changes and problems with embryo implantation in women struggling with infertility. Specifically, we looked at variations in the number of copies of certain genes, CNVs, and how they relate to a protein called integrin β3 in the lining of the uterus. About one-third of the women we studied had these genetic variations, which were mostly found on chromosomes. We discovered that older women were more likely to have these CNVs, especially those with repeated implantation failure. Furthermore, women with these genetic changes had higher levels of integrin β3 in their uterine lining compared to those without the changes. We also found that these genetic variations were linked to differences in blood platelet counts and blood clotting times, suggesting a possible impact on blood coagulation. Our findings shed light on how genetic changes might influence both the uterine environment and blood clotting, potentially affecting a woman’s ability to conceive. This knowledge could help in developing personalized treatments for women facing recurrent implantation failure, improving their chances of successful pregnancy.

## Introduction

Infertility is a serious condition that significantly affects the physical and mental health of patients and is a major concern for both the medical community and society. The rapid development of assisted reproductive technology (ART) has provided new possibilities for treating infertility. However, clinical pregnancy rates have exhibited fluctuations despite the increased chances of obtaining high-quality embryos during treatment cycles. In particular, embryo implantation failure has become the leading cause of ART treatment failure (Szamatowicz 2016, De 2019, Esteves *et al.* 2019, Seli & Garcia-Velasco 2021). Some patients still struggle to achieve pregnancy after multiple transfers of high-quality embryos despite ruling out factors such as uterine cavity abnormalities and chromosomal abnormalities. This condition is known as recurrent implantation failure (RIF), affecting approximately 10% of patients undergoing ART (Lessey *et al.* 2006, Coughlan *et al.* 2014).

RIF exhibited multifactorial causes, including uterine abnormalities, genetic factors and endometrial receptivity problems. Integrin αvβ3, an adhesion molecule receptor, plays a crucial role in the endometrium when investigating the mechanisms behind RIF. Integrin αvβ3 is an adhesion molecule expressed in the endometrium that facilitates embryo attachment and invasion into the endometrial stroma, thereby playing a crucial role in the implantation process. Integrin αvβ3 connects the extracellular matrix and the cell cytoskeleton to mediate bidirectional recognition, adhesion and implantation of the embryo in the endometrium. Studies have revealed that reduced expression or dysfunction of integrin αvβ3 negatively affects endometrial receptivity and causes implantation failure. Notably, recent studies have demonstrated differences in integrin αvβ3 expression between individuals with successful and failed implantation, indicating its potential as a biomarker for endometrial receptivity (Thornhill *et al.* 2005, Achache & Revel 2006, Ziegler & Buckner 2009, Johnston-MacAnanny *et al.* 2010, Granot *et al.* 2012). In addition, gene copy number variations (CNVs) have appeared as significant factors affecting gene expression and phenotypic outcomes. CNVs involve genomic region duplications or deletions, which alter the expression of genes that are crucial for physiological processes, including reproduction. Recent research has revealed that CNVs affect the expression levels of genes that are associated with endometrial receptivity and fertility, thereby further emphasizing the necessity of investigating the role of CNVs in RIF.

This study hypothesizes that CNVs may affect the expression of integrin αvβ3 in the endometrium, thereby causing implantation failure. This study aims to elucidate the association between CNVs and integrin αvβ3 expression by analyzing endometrial tissues from patients with RIF and comparing them with controls (Henrichsen *et al.* 2009, Ionita-Laza *et al.* 2009, Gamazon & Stranger 2015, Pös *et al.* 2021). Understanding this association will help to gain deeper information into the molecular mechanisms of RIF, thereby providing new perspectives and approaches for infertility treatment. Ultimately, this comprehensive study aims to provide more targeted treatment strategies for patients with RIF and improve the success rate of ART treatments.

## Materials and methods

### Participants

The Ethics Committee of The First People’s Hospital (Yunnan Province) approved this study, which collected endometrial samples from 48 patients with RIF (>2 failures) and ten patients with infertility who had not undergone any embryo implantation procedures. All participants provided informed consent. Hormone therapy administration before biopsy was recorded for patients with RIF. Biopsies were conducted during the proliferative phase and the window of implantation (WOI). Endometrial tissue was collected on days 12 (proliferative phase) and 21 (WOI) of the natural menstrual cycle before the *in vitro* fertilization–embryo transfer cycle. WOI was identified based on a combination of cycle days, hormone levels and ultrasonographic evaluation of endometrial thickness and pattern. Both biopsies (proliferative phase and WOI) were conducted within the same menstrual cycle to ensure consistency. Endometrial biopsies were obtained using the endometrial sampling tube technique from three different locations within the uterine cavity, including the fundus and sides, divided into triplicates and preserved in saline, formalin and DNA/RNA shield for subsequent protein blotting, immunohistochemistry, real-time quantitative polymerase chain reaction (PCR) and CNVs.

The inclusion criteria were as follows. RIF infertility group (48 cases): i) patients aged ≤38 years, ii) number of IVF cycles, with experience of at least three embryo transfer cycles, with no pregnancy achieved in each cycle where 1–2 high-quality embryos were transferred and iii) embryo availability, with at least two cryopreserved embryos (cleavage-stage or blastocyst-stage), including at least one high-quality embryo. Unexplained infertility group (ten cases): i) normal basal endocrine levels, ii) regular menstrual cycles, iii) no history of uterine procedures or hormone use within the past 3 months and iv) not currently pregnant. RIF group: i) presence of uterine malformation, history of submucosal fibroid removal or intrauterine adhesions, ii) diagnosis of pelvic endometriosis, ovarian endometriotic cysts or adenomyosis, iii) undergoing advanced ART procedures, including intracytoplasmic sperm injection, preimplantation genetic diagnosis or pre-implantation genetic screening before embryo transfer, iv) presence of metabolic disorders, infections, thrombosis tendencies or antiphospholipid antibody syndrome and v) retrieval of <2 eggs or <2 frozen embryos. Unexplained infertility group: i) abnormal uterine morphology, ii) diagnosis of uterine endometriosis, iii) presence of active infectious diseases and iv) diagnosis of severe hydrosalpinx.

### Reagents

The following reagents were used in this study: Trizol (266411, Thermo Fisher, USA); PowerUP SYBR Green Master Mix (A25742, Thermo Fisher); RIPA buffer (high) (R0010, Solarbio, China); BCA Protein Assay Kit (PC0020; Solarbio, CA); anti-integrin beta antibody (ab210515, Abcam, USA); GAPDH antibody (ab181602; Abcam, USA); secondary antibody (IgG H&L) (ab6721, Abcam, USA). All PCR experiments were performed according to the ‘Minimum Information for the Publication of Real-Time Quantitative PCR Experiments’ (MIQE) guidelines (Bustin *et al.* 2009).

### CNV detection

DNA was extracted from 58 endometrial tissue samples obtained from patients with RIF and unimplanted infertility using the QIAamp DNA Mini Kit, following the manufacturer’s protocol. The quality of the extracted DNA was assessed using two methods: i) agarose gel electrophoresis to evaluate DNA integrity and check for RNA contamination and ii) the Qubit fluorometer with the Qubit dsDNA HS Assay Kit to accurately quantify DNA concentration. Only samples with minimal degradation, no RNA or small fragment contamination, and a clear appearance without precipitation were selected for further processing. The DNA concentration had to be at least 20 ng/μL and a total of 1 μg of DNA was required for subsequent library preparation.

Genomic DNA libraries were constructed following a series of steps. First, the DNA was enzymatically fragmented to target sizes, followed by end repair and A-tailing to prepare for adapter ligation. Next, adapters were ligated to the DNA fragments to create the library structure. After ligation, the libraries were amplified via PCR, purified and eluted. To ensure library quality, several steps were taken: i) initial quantification using the Qubit 4.0 fluorometer, ii) insert size detection using the Agilent 2100 Bioanalyzer and iii) final concentration measurement by qPCR to ensure an effective library concentration of 2 nM.

High-throughput sequencing was performed on the Illumina NovaSeq 6000 platform in PE150 mode, generating approximately 5 million sequencing reads of 36 base pairs each. Of these, 2.8 to 3.2 million reads were uniquely mapped to the human genome (hg19) using the Burrows-Wheeler algorithm. The mapped reads were distributed into 20-kilobase (kb) bins across all chromosomes, with read counts in each bin compared across samples to detect CNVs based on previously established algorithms. Chromosome profiles were generated, showing copy number against 20 kb count windows, with a blue line indicating the mean copy number to help identify regions of deletion or duplication.

Identified CNVs were cross-referenced against publicly available databases such as Decipher, the Database of Genomic Variants (DGV), 1000 Genomes and Online Mendelian Inheritance in Man (OMIM). Pathogenicity was assessed following guidelines from the American College of Medical Genetics (ACMG) for sequence variant interpretation. CNVs were categorized as pathogenic, likely pathogenic, variants of uncertain significance (VUS), likely benign or benign. For this study, only pathogenic, likely pathogenic and VUS were considered in the final analysis. When CNVs demonstrated variable penetrance or expressivity, they were classified as likely pathogenic.

The burden of CNVs was evaluated by calculating the total number of CNVs per sample, their cumulative length, and their distribution across functionally significant genomic regions. Only CNVs deemed clinically relevant (pathogenic, likely pathogenic or VUS) were included in this burden assessment to estimate the overall impact of these CNVs on genomic integrity.

### Real-time quantitative PCR detection

PCR primers for integrin αvβ3 were designed, and Table 1 presents the primer sequences, with glyceraldehyde 3-phosphate dehydrogenase (GAPDH) utilized as an internal reference. This study analyzed 58 endometrial samples from patients with repeated implantation failure and infertility without implantation. The samples were lysed with Trizol, extracted with chloroform (10,957 ***g***, 15 min, 4°C), precipitated with isopropanol (8,050 ***g***, 10 min, 4°C), washed with 75% ethanol (5,590 ***g***, 5 min, 4°C), and the RNA of the samples was obtained. The purity and concentration of the sample RNA were identified with the NanoDrop 2000. Takara’s reverse transcription kit was then utilized according to its instruction manual to reverse transcribe the sample RNA into cDNA. The Thermo Fisher SYBR kit was used for qPCR experiments with cDNA as a template. Tables 2 and 3 present the qPCR reagents and reaction system.

**Table 1:** Integrin αvβ3 PCR Primer Sequences.

Gene	Primer	Sequence (5′-3′)		Amplicon size (bp)
		Forward	Reverse	
*ITGAV*	αV	cgt​atc​tgc​ggg​atg​aat​ct	ggg​ttg​caa​gcc​tgt​tgt​at	110
*ITGB3*	β3	tgg​tcc​tgc​tct​cag​tga​tg	gaa​ttc​ttt​tcg​gtc​gtg​ga	98
*GAPDH*	GAPDH	cca​ccc​aga​aga​ctg​tgg​at	ttc​agc​tca​ggg​atg​acc​tt	127

ITGAV, integrin subunit alpha V; ITGB3, integrin subunit beta 3; PCR, polymerase chain reaction.

**Table 2:** qPCR reagents.

Reagent	Utilization
PowerUP SYBR green master mix	10 μL
PCR forward primer	1 μL
PCR reverse primer	1 μL
ddH2O	5 μL
cDNA	2 μL
Total	20 μL

PCR, polymerase chain reaction.

**Table 3:** qPCR reagent reaction systems.

Condition	Temperature	Time	Cycles, *n*
UDG activation	50°C	2 min	
DNA polymerase	95°C	2 min	
Denature	95°C	3 s	40
Anneal/extend	60°C	30 s	

### Protein immunoblotting detection

Endometrial tissues were collected from patients with RIF (≥2 times) and infertility with no implantation. Radioimmunoprecipitation assay lysis buffer (phenylmethylsulfonyl fluoride = 100:1) was added to the tissues, ground on ice, incubated the samples on ice for 30 min, and centrifuged at 8,050 ***g*** for 10 min to collect the supernatant. The protein content in the samples was measured with the BCA protein quantification method. The samples were diluted to 1× with 5× protein loading buffer, boiled at 95°C for 10 min, and immediately placed on ice. Samples and molecular weight markers were loaded onto sodium dodecyl sulfate-polyacrylamide gel electrophoresis with an amount of 200 ng per well, and electrophoresis was performed (80 V for 30 min followed by 120 V for 35 min). Proteins were transferred from the gel to a polyvinylidene fluoride membrane with a semi-dry transfer method. The membrane was blocked with 5% BSA on a shaker at room temperature for 2 h. The primary antibodies (integrinβ3 and GAPDH) were diluted to an appropriate concentration following the antibody instructions, and the membrane was incubated overnight at 4°C with shaking. The membrane was washed five times with Tris-buffered saline with Tween-20 (TBST), each time for 5 min. Secondary antibodies were diluted to an appropriate concentration, and the membrane was incubated at room temperature for 1 h with shaking. The membrane was washed five times with TBST, each time for 5 min. The membrane was then placed in a chemiluminescence imager and developed, and ImageJ software was used for grayscale analysis to compare protein levels among different groups.

### Tissue section staining

Endometrial tissues were retrieved from patients with RIF (≥2 times) and infertility with no implantation and stored in formalin. The tissues were dehydrated in a series of alcohol solutions with increasing concentrations, followed by xylene for transparency, and then embedded in paraffin. The embedded endometrial tissues were sectioned into thin slices of 5–8 micrometers with a microtome. The slices were flattened by heating them in warm water and affixing them to glass slides. The slides were dried in a 45°C constant-temperature incubator. After dewaxing, the sections were stained with hematoxylin and eosin (HE), dehydrated, cleared and observed under an optical microscope.

### Immunohistochemistry detection

Endometrial tissues were retrieved from patients with RIF (≥2 times) and infertility with no implantation and stored in formalin. The tissues were dehydrated in a series of alcohol solutions with increasing concentrations, followed by xylene for transparency, and then embedded in paraffin. The embedded endometrial tissues were sectioned into thin slices of 5–8 micrometers with a microtome. The slices were flattened by heating them in warm water and affixing them to glass slides. The slides were dried in a 45°C constant-temperature incubator. The sections were dewaxed at room temperature, hydrated, performed antigen retrieval and were outlined for immunohistochemistry, endogenous enzyme activity was deactivated, and blocked with goat serum at room temperature for 10 min. The primary antibody (polyclonal rabbit anti-human integrin β3) was applied, diluted at 1:200, and incubated overnight at 4°C. The secondary antibody was applied, 3,3′-diaminobenzidine color development was performed, counterstained with hematoxylin, dehydrated, cleared and mounted with neutral resin. The expression difference of integrin β3 was observed in the endometrium under an optical microscope.

### Statistical analysis

Statistical analyses were performed using SPSS version 22.0 (IBM Corp, USA). A two-way ANOVA was used to assess the interaction effects between CNV status and experimental conditions (unimplanted vs IVF-ET failure) on the expression levels of integrin αV and β3. Post-hoc tests with Bonferroni correction were applied to account for multiple comparisons. Independent sample *t*-tests were conducted between specific groups, and *P*-values were adjusted using the Holm–Bonferroni correction. For categorical data, the χ^2^ test was used, while t-tests were applied for quantitative data, presented as mean ± standard deviation (x ± SD). Statistical significance was considered at *P* < 0.05.

## Results

### Gene CNVs in the endometrium of patients with RIF and infertility with no implantation

Chromosomal aneuploidy and CNV detection were performed on endometrial tissues from 58 patients, including 48 with RIF and ten with infertility with no history of implantation issues. The data analysis revealed that among the 58 samples, 18 (31.04%) demonstrated CNVs (clinical significance unclear), whereas 40 (68.96%) samples exhibited no detected CNVs. This indicates that close to 1/3 of patients with infertility experience CNVs. In addition, a detailed analysis of the 18 cases with detected CNVs revealed that 15 cases exhibited single-chromosome abnormalities, and three demonstrated two-chromosome abnormalities. The chromosomes most predominantly affected were 2, 5, 6, 7, 10, 11, 15, 19 and X. The types of variations included duplications and deletions. Tables 4 and 5 show specific details on variation locations, fragment sizes and corresponding IVF-ET failure frequencies.

**Table 4:** Single chromosome aberration.

Cases, *nl*Location of variation	Type of variation	Chromosome	Fragment size, Mb	IVF-ET failures, *n*
3	Replicate	X		
q25			0.56	6
p21.1			0.24	5
p11.3			0.50	5
3	Replicate	2		
p24.1			0.28	3
q11.1q11.2			0.94	/
p12p12			0.18	/
1 p13.1p13.1	Replicate	5	0.14	2
1 q22.31q22.31	Missing	6	0.40	/
1 q21.11	Replicate	7	0.50	4
1 q21.1q21.1	Missing	10	0.24	2
2 p15.1	Replicate	11	0.70	4

**Table 5:** Two chromosome aberrations.

Sample/chromosome	Type of variation	Location of variation	Fragment size, Mb	IVF-ET failures, *n*
1				2
5	Replicate	p15.33p15.33	0.30	
18	Replicate	q23q23	0.28	
2				2
7	Replicate	p22.2p22.2	0.34	
11	Replicate	q25q25	0.16	
3				-
19	Missing	p12p12	0.42	
18	Replicate	p11.31p11.23	0.48	

Furthermore, statistical analysis was conducted on the occurrence of CNVs and the age of the RIF and unimplanted groups. The unimplanted group exhibited an average age of 34.38 ± 4.37 years, with a CNV detection positive rate of 50.00%. The RIF group (IVF-ET failures) demonstrated an average age of 34.31 ± 4.16 years, with a CNV detection positive rate of 37.14%. This indicates that the ages of patients with infertility generally range from 34 to 39 years and that the occurrence rates of CNV variations are relatively high. Subsequent grouping analysis of the RIF group (IVF-ET failures) based on different numbers of failures revealed (Table 6). Patients with 2, 3, 4 and 5 times IVF-ET failures demonstrated an average age of 37.2 ± 1.67, 32 ± 4.01, 37 ± 3.04 and 35 ± 3.74 years, with CNV detection positive rates of 55.56, 10.50, 16.70 and 50%, respectively. This indicates that the occurrence rates of CNVs in patients with RIF of ≥2 times are ≥50%, and their ages generally range from 35 to 38 years. Conversely, the CNV occurrence rates for those with RIF of 3 and 4 times are relatively lower. Among them, patients with IVF-ET of 3 times exhibited significantly lower ages than the other groups, ranging from 32 to 36 years. This result indicates no proportional relationship between the CNV occurrence rate and the number of RIFs, but with a correlation. Moreover, CNVs are more likely to occur in older individuals, whereas the CNV occurrence rate is lower in younger individuals.

**Table 6:** Group analysis of different numbers of RIF.

Group	*n*	Age* (years)	CNVs detection positive rate
Unimplanted	10	34.38 ± 4.37	50.00%
IVF-ET failures	48	34.31 ± 4.16	37.14%
IVF-ET failures, *n*			
2	9	37.2 ± 1.67	55.56%
3	19	32 ± 4.01	10.50%
4	12	37 ± 3.04	16.70%
≥5	8	35 ± 3.74	50.00%

RIF, recurrent implantation failure.

* Values are mean ± SD.

### Nucleic acid expression of integrin αVβ3 in the endometrium of the unimplanted and RIF groups: evaluation and comparison of the correlation between CNV occurrence and αVβ3 gene differential expression

**Figure 1:**
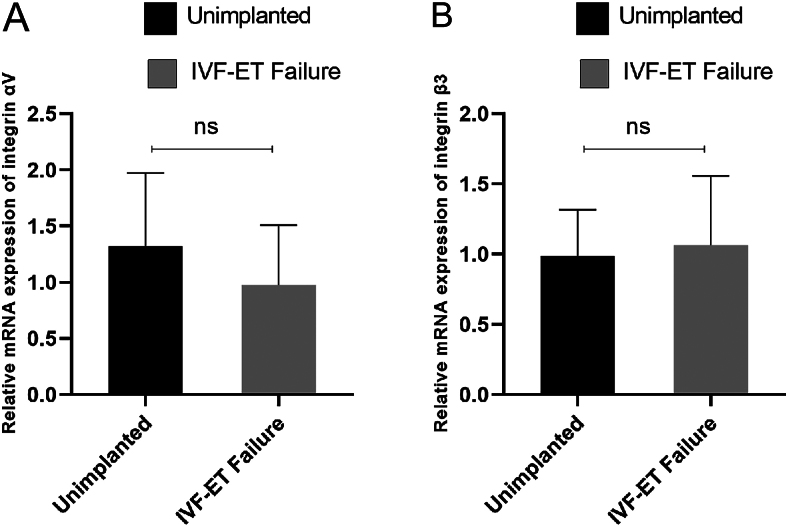
Comparison of the nucleic acid expression of integrin αVβ3 in the endometrium between the two groups of patients. (A) The expression of αV is higher in the non-implanted group compared to the group with repeated implantation failure. (B) β3 expression is lower in the non-implanted group compared to the group with repeated implantation failure. No statistically significant difference. IVF-ET failure, *in vitro* fertilization-embryo transfer failure.

Real-time qPCR was conducted to investigate the nucleic acid expression of integrin αVβ3 in the endometrium of the unimplanted and RIF groups. The results (Fig. 1) revealed that αV expression in the unimplanted group was higher than that in the RIF group, whereas β3 expression in the unimplanted group was lower than that in the RIF group, with no statistically significant differences.

**Figure 2:**
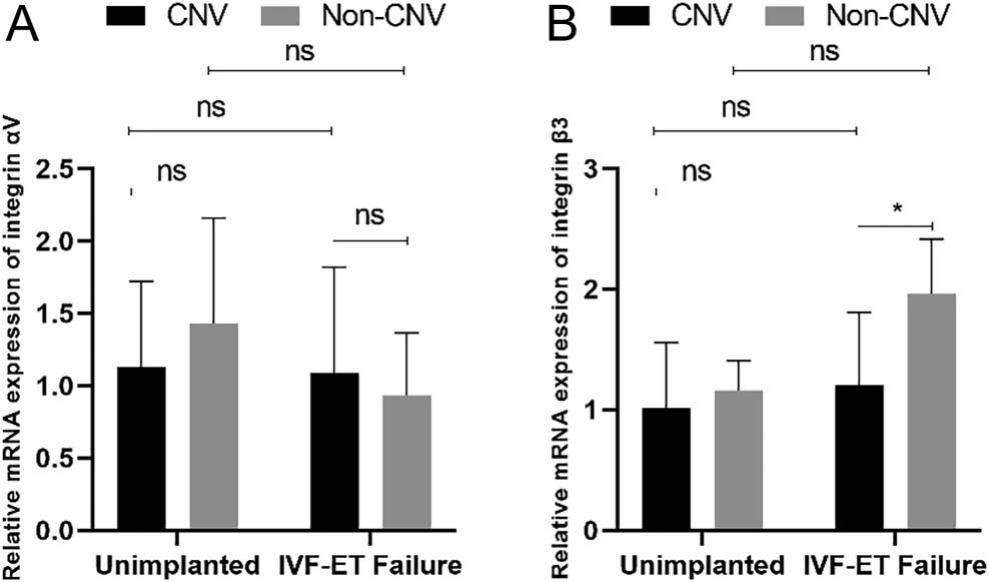
Comparison of nucleic acid expression of integrin αVβ3 between two patient groups, one with CNVs and the other without. (A) The nucleic acid expression of integrin αV in the endometrium shows no significant difference between the two patient groups, one with CNVs and the other without. (B) In the IVF-ET failure group, there is a significant difference in the nucleic acid expression of β3 in the endometrium between patients with CNVs and those without, with the CNV group significantly higher than the non-CNV group. However, in the non-implanted group, there is no significant correlation between the nucleic acid expression of integrin β3 and the occurrence of CNVs. *P < 0.05. CNVs, copy number variations. IVF-ET failure, *in vitro* fertilization-embryo transfer failure.

Subsequently, based on whether CNVs occurred in all patients mentioned above, we categorized the unimplanted and RIF groups into the CNV and non-CNV groups. The statistical data (Fig. 2) revealed no significant difference in the nucleic acid expression of integrin αV in the endometrium between those with CNVs and those without CNVs, whether in the unimplanted or RIF group. However, the nucleic acid expression of integrin β3 was significantly different between patients with CNVs and those without CNVs in the RIF group, with the CNV group significantly higher than the non-CNV group. No significant correlation between the nucleic acid expression of integrin β3 and the occurrence of CNVs was found in the unimplanted group. This result suggests a correlation between CNV occurrence in patients with repeated implantation failure and elevated integrin β3 expression. This is inconsistent with many previous studies reporting low αVβ3 expression in patients with infertility. Therefore, we further searched the relevant literature and revealed that these studies did not test patients’ endometrial samples for CNVs (Lessey 1994, Cavagna & Mantese 2003, Franasiak *et al.* 2014*b*, Gnainsky *et al.* 2015, Elnaggar *et al.* 2017). Our study introduces advanced molecular diagnostic technology, and we further searched for the function of integrin β3 in the literature based on this result and revealed its close association with thrombosis (Bennett *et al.* 1997). Therefore, we hypothesize that the high integrin β3 expression may contribute to a risk of intrauterine thrombosis formation, which could be associated with repeated implantation failure, particularly when CNVs occur in the endometrium of patients with repeated implantation failure.

Moreover, we retrospectively investigated the coagulation function of 33 of these patients with recurrent implantation failures, involving relevant clinical tests (white blood cell count, absolute neutrophil count, absolute lymphocyte count, absolute monocyte count, absolute eosinophil count, absolute basophil count, erythrocyte count, hemoglobin volume, platelet count, activated partial thromboplastin time, prothrombin time, international normalized ratio, thrombin time, fibrinogen content (FIB), prothrombin time activity (PTTA) and D-dimer values) were statistically analyzed based on the above innovative findings. The results revealed that the platelet count and PTTA in the CNV group of patients with RIF were significantly lower than those in the non-CNV group (Table 7), but the platelet and PTTA values in both groups were within the normal range. This indicates that the significant reduction in these two indicators, still within the normal reference range, is closely associated with CNV occurrence in patients with repeated implantation failure.

**Table 7:** Clinical diagnostic indicators in patients with RIF.

Indicator	CNVs (*n* = 5)	Non-CNVs (*n* = 28)	*P* values
White blood cell count	5.65 ± 1.65	6.96 ± 1.75	0.13
Absolute neutrophil count	3.33 ± 1.31	4.08 ± 1.40	0.27
Absolute lymphocyte count	1.87 ± 0.41	2.26 ± 0.84	0.32
Absolute monocyte count	0.40 ± 0.10	0.44 ± 0.14	0.49
Absolute eosinophil count	0.04 ± 0.02	0.13 ± 0.12	0.08
Absolute basophil count	0.03 ± 0.01	0.04 ± 0.02	0.19
Red blood cell count	4.74 ± 0.09	4.83 ± 0.44	0.66
Hemoglobin level	143.40 ± 5.59	142.57 ± 9.25	0.85
Platelet count	231.0 ± 50.03	289.35 ± 50.73	0.02
APTT (s)	34.83 ± 2.08	33.95 ± 5.58	0.79
PT (s)	12.47 ± 0.25	11.63 ± 0.83	0.11
INR	0.93 ± 0.01	0.91 ± 0.04	0.39
TT (s)	17.80 ± 1.35	18.42 ± 0.91	0.33
Fibrinogen level (g/L)	3.16 ± 0.47	3.22 ± 0.0.70	0.88
PTTA (%)	113.0 ± 1.73	121.15 ± 10.93	0.01
DD2 (μg/mL)	0.86 ± 0.28	0.76 ± 0.33	0.59

PTTA, prothrombin time activity; DD2, D-dimer; RIF, recurrent implantation failure.

### Protein immunoblotting and immunohistochemistry for integrin β3 expression in the CNV and non-CNV groups: comparative analysis 

**Figure 3:**
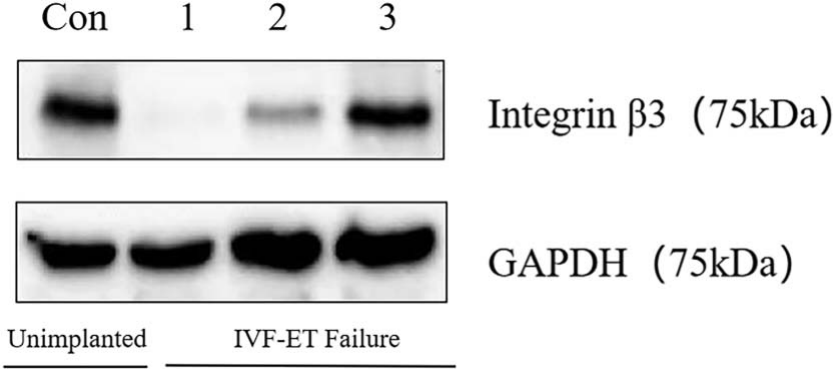
The protein expression of integrin β3 in the endometrium of patients from the non-implanted group and the group with IVF-ET failure was assessed by western blot. The protein level of β3 in the endometrium of patients without implantation and non-CNVs is higher, while in patients with IVF-ET failure and CNVs, the protein level of β3 is higher compared to those non-CNVs in the group with IVF-ET failure. Con, healthy population. 1, IVF-ET failed twice, no CNVs detected. 2, IVF-ET failed twice, CNV: seq[hg19]dup(7)(q35q35)chr7:g.143420000_143840000dup. 3, IVF-ET failed twice, CNV: seq[hg19]dup(17)(q21.32q21.32)chr17:g.46400000_46760000dup. CNVs, copy number variations. IVF-ET failure, *in vitro* fertilization – embryo transfer failure.

**Figure 4:**
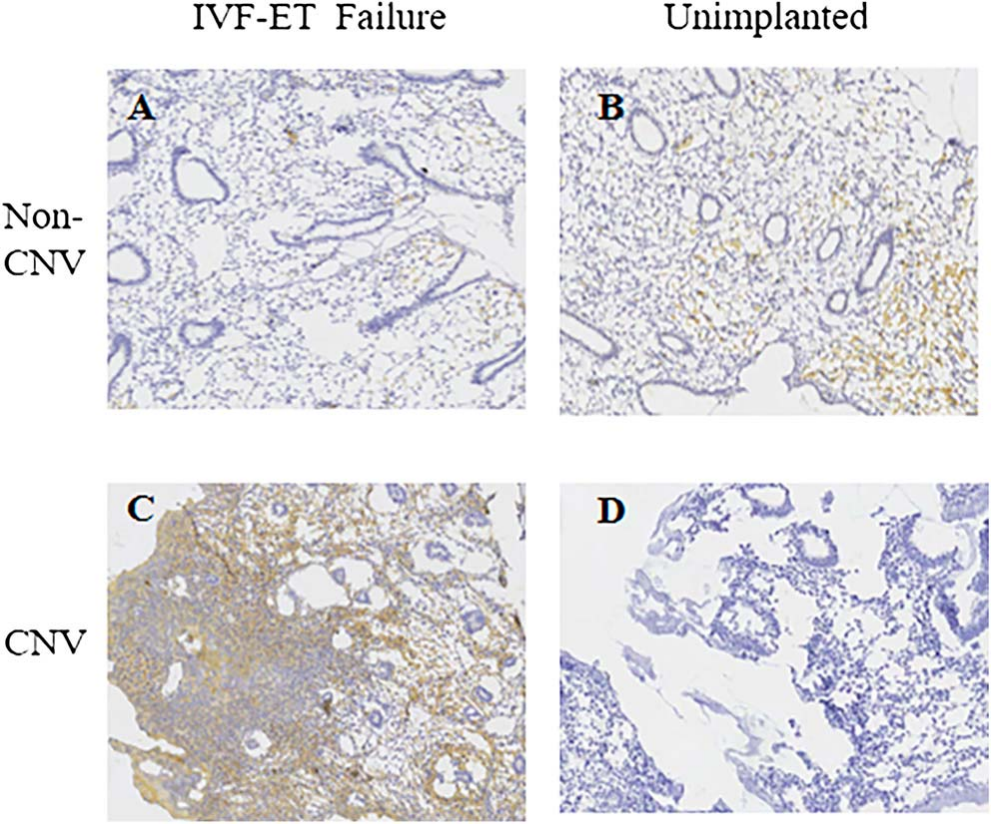
Immunohistochemistry. Integrin β3 expression: comparison of β3 immunostaining between CNV and non-CNV patients in the IVF-ET failure group and CNV and non-CNV patients in the unimplanted group, (A and C) in the IVF-ET failure group, in the IVF-ET failure group, CNV patients were strongly positive for integrin β3 expression than non-CNV patients; (B and D) in the unimplanted group, the integrin β3 showed weak positive staining in both CNV patients and non-CNV patients. Magnification 40×, the scale bars in the figures indicate 200 μm. CNVs, copy number variations. IVF-ET failure, *in vitro* fertilization – embryo transfer failure.

After CNV detection, protein immunoblotting and immunohistochemistry experiments were separately conducted on the endometrial integrin β3 of patients in the CNV and non-CNV groups. The results revealed (Figs 3 and 4) that the endometrium of patients without implantation and without CNVs exhibited lower β3 protein levels, whereas both β3 proteins of patients with repeated implantation failure and CNVs were significantly higher than those with repeated implantation failure without CNVs. These protein results were consistent with nucleic acid results, thereby further confirming our previous hypothesis that elevated integrin β3 expression when CNVs occur in the endometrium of patients with repeated implantation failure may promote intrauterine thrombosis formation, causing repeated implantation failure. This finding may offer insights into potential pathogenic factors in certain patients with infertility.

### Comparative analysis of HE staining on tissue slices 

**Figure 5:**
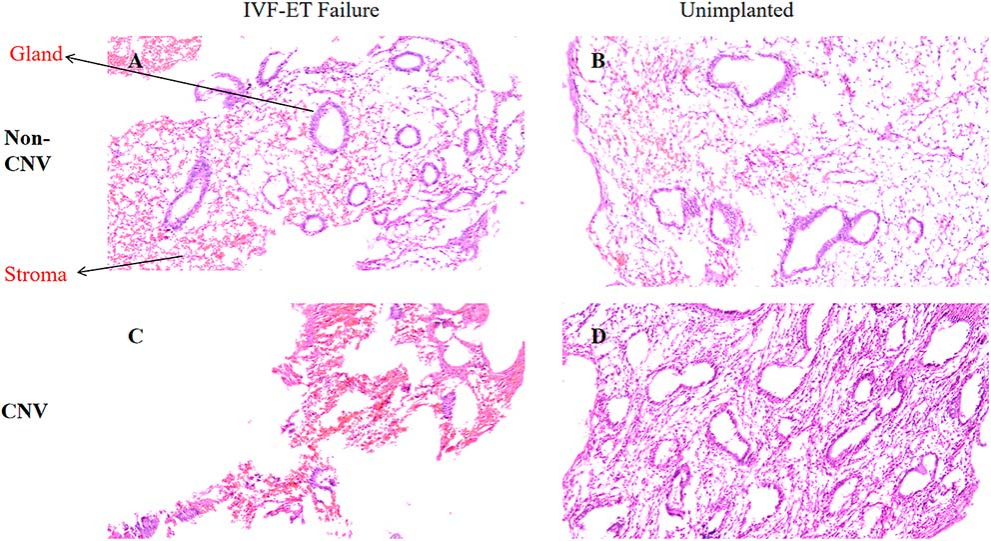
HE staining. Morphological observation of endometrial tissue sections: the results from both CNV and non-CNV patients are presented in Figures (A and C) for the repeated implantation failure group and (B and D) for the unimplanted group. Magnification 100×, the scale bars in the figures indicate 100 μm.

Tissue slices of the endometrium from patients with repeated implantation failure and CNVs were investigated through HE staining to observe morphological changes in the relevant components. The observations revealed the following (Fig. 5). Patients with CNVs and repeated implantation failure demonstrated irregular and curved glandular morphology. No subnuclear or supranuclear vacuoles were observed under the glandular epithelium. The interstitial region demonstrated edema, fibrosis and severe hemorrhage, indicating that these histological features may be associated with implantation failure. Patients with CNVs in the unimplanted group exhibited glands with more normal morphology, normal interstitial cells and structures, and slight edema. Patients with non-CNVs and repeated implantation failure demonstrated relatively regular glandular morphology with slight glandular maldistribution and increased interstitial density. Patients with non-CNVs in the unimplanted group showed irregular glandular and interstitial structures, with interstitial edema.

These results indicate that repeated implantation failures in patients with CNVs may be associated with tissue edema and hemorrhage. These histological features may be contributing factors influencing the success rate of IVF-ET.

In summary, our investigation into gene CNVs in the endometrium of patients with RIF and infertility with no implantation revealed intriguing results. Approximately one-third of patients with infertility exhibited CNVs, with specific abnormalities observed in chromosomes 2, 5, 6, 7, 10, 11, 15, 19 and X. Age appeared to correlate with CNV occurrence, particularly in patients with RIF. In addition, our study investigated integrin αVβ3 expression and revealed significant differences in β3 expression associated with CNVs in the RIF group. Furthermore, analyses related CNV occurrence to variations in platelet count and prothrombin time activity, indicating potential implications for coagulation function. In conclusion, these results provide valuable insights into the complex association between CNVs, integrin expression and coagulation function in the context of RIF, though further studies are necessary to establish causal relationships. Understanding these molecular and clinical aspects may help guide the development of personalized reproductive medicine interventions. This research highlights the importance of comprehensive assessments in infertility cases and underscores the potential role of CNVs and integrin β3 in the pathogenesis of RIF. Our study contributes to a better understanding of molecular mechanisms and highlights the need for tailored approaches in assisted reproductive technologies, acknowledging the complexities of reproductive health. Moving forward, integrating these results into clinical practice could improve diagnostic precision and therapeutic strategies, ultimately enhancing outcomes for individuals who face challenges in achieving successful implantation and pregnancy.

## Discussion

Our study revealed, for the first time, that integrin β3 was abnormally expressed in the endometrium of patients with repeated implantation failures after CNV detection. CNVs were detected in 18 of 58 patients, although the clinical significance of this finding remains unclear. We observed that different CNV variations caused various integrin β3 expressions even in patients with repeated implantation failures. We hypothesize that this is potentially associated with thrombosis in the endometrial tissue, which hinders embryo implantation.

In addition, we observed that high β3 integrin levels were associated with repeated embryo implantation failure. This result initially appeared to be somewhat counterintuitive, as β3 integrin is frequently considered an endometrial receptivity marker. However, upon further reflection, we realized that elevated β3 integrin levels may reflect a ‘post-receptive’ state in which the endometrium has passed its optimal implantation window, making it less supportive of successful implantation. This hypothesis is congruent with the broader concept of endometrial receptivity being a tightly regulated window, revealing that elevated β3 integrin levels may indicate that the endometrium has exceeded its optimal receptivity period, causing embryo implantation failure. This new perspective provides a basis for further investigation of the association between endometrial receptivity and β3 integrin expression.

Our study shows, for the first time, the non-classical function of β3 in integrin αvβ3, which is associated with thrombosis in patients with infertility, and explores a key reason for RIF in such patients. Meanwhile, the application of integrin β3 expression combined with CNV detection to evaluate endometrial receptivity in patients with repeated implantation failure has significant clinical guidance value and potential for clinical translational application. This study is highly innovative and has substantial value for clinical promotion and application compared with similar research conducted domestically and internationally.

While our findings provide novel insights, there are several important limitations that must be acknowledged. First, the sample size in this study is relatively small (*n* = 58), limiting the statistical power to detect more subtle relationships between CNVs and integrin β3 expression. Larger cohort studies are needed to confirm these associations and further clarify the relationship between CNVs and endometrial receptivity. Second, the clinical relevance of the detected CNVs remains unclear, as our study does not definitively establish causality between CNVs and RIF. Although we observed an association, the underlying biological mechanisms require further elucidation, possibly through functional studies or animal models.

Another limitation is the lack of longitudinal data, which would allow us to examine how CNV status and integrin β3 expression fluctuate across different menstrual cycles and their potential role in predicting implantation success in subsequent IVF-ET cycles. Furthermore, this study did not account for potential confounding factors such as hormonal levels, lifestyle factors and other genetic variations that could influence endometrial receptivity and CNV occurrence.

Our results align with previous studies suggesting that age is a risk factor for CNV development, as we found CNVs to be more prevalent in older patients. This observation is consistent with previous research indicating genomic instability and decreased DNA repair capacity as potential contributors to the age-related increase in CNV frequency. For example, studies by Franasiak *et al.* (2014*a*,*b*) and Cakmak and Taylor (2011) have also reported a higher frequency of chromosomal abnormalities in older individuals undergoing IVF treatment (Cakmak & Taylor 2011, Franasiak *et al.* 2014*a*). However, unlike these studies, our research uniquely highlights the specific relationship between CNV presence and abnormal integrin β3 expression, offering new insights into the molecular mechanisms of RIF.

In addition, our findings challenge the traditional view that elevated integrin β3 levels are universally indicative of increased endometrial receptivity. As noted in studies such as Diedrich *et al.* (2007) and Johnson *et al.* (2023), integrin β3 has often been used as a marker of endometrial receptivity, but our results suggest that its elevated expression in patients with CNVs may signify a post-receptive state rather than enhanced receptivity, further complicating its role in reproductive diagnostics (Diedrich *et al.* 2007, Johnson *et al.* 2023).

The clinical implications of our findings are substantial, particularly in the context of improving diagnostic accuracy for patients with RIF. CNV detection combined with integrin β3 expression analysis may provide a more comprehensive assessment of endometrial receptivity and help identify patients who are at higher risk for implantation failure due to undiagnosed genomic abnormalities. This could lead to more personalized treatment strategies, such as adjusting the timing of embryo transfer to align with the endometrium’s optimal receptivity window.

Moving forward, future research should focus on expanding the scope of case collection and developing animal models to further investigate the role of CNVs in endometrial function and integrin β3 expression. This will help validate our findings and clarify the non-classical function of integrin β3 in the context of thrombosis and implantation failure. In addition, longitudinal studies that track patients across multiple IVF cycles could provide deeper insights into the dynamic relationship between CNVs, integrin expression and implantation outcomes.

In conclusion, while our study sheds light on the potential role of CNVs and integrin β3 in the pathogenesis of RIF, it also underscores the need for further research to elucidate the underlying mechanisms and optimize clinical interventions. Understanding these molecular pathways could pave the way for more targeted and effective treatments, ultimately improving reproductive outcomes for patients facing repeated implantation failures.

## Declaration of interest

The authors declare that there is no conflict of interest that could be perceived as prejudicing the impartiality of the work reported.

## Funding

The work was supported by the Science and Technology Department of Yunnan Province Joint Special Project (202301AY070001-227), Major Science and Technology Program of Yunnan Province (202302AA310044), Yunnan Provincial Department of Science and Technology Special Programme for Selection of High-level Scientific and Technological Talents and Innovation Teams (202105AC160012) and Key Project of Basic Research, Yunnan Provincial Science and Technology Department (202401AS070008).

## Author contribution statement

Li Li participated in study design, results analysis and interpretation, manuscript preparation, data collection, data processing and statistical analysis. Hongxia Xu, Shengni Liu and Xiaoyi Xiang participated in some data collection and processing. Yan Lei, Yunxiu Li, Xiangjing Tang, Jianmei Yu, Limei Tao and Ze Wu participated in sample collection and processing. All authors read and approved the final manuscript.

## Data availability

The datasets used and analyzed during the current study are available from the corresponding author upon reasonable request.

## Ethics approval and consent to participate

The human tissue samples involved in this study were obtained from the Department of Reproductive Medicine of the First People’s Hospital of Yunnan Province. Our study was a reuse of clinical samples. The Ethics Committee of the First People’s Hospital of Yunnan Province approved the informed consent for this study.

## References

[bib1] Achache H & Revel A 2006 Endometrial receptivity markers, the journey to successful embryo implantation. Hum Reprod Update 12 731–746. (10.1093/humupd/dml004) 16982667

[bib2] Bennett C, Vilaire M & DeGrado 1997 Agonist-activated alphavbeta3 on platelets and lymphocytes binds to the matrix protein osteopontin. J Biol Chem 272 8137–8140. (10.1074/jbc.272.13.8137) 9079626

[bib3] Bustin SA, Benes V, Garson JA, et al. 2009 The MIQE guidelines: minimum information for publication of quantitative real-time PCR experiments. Clin Chem 55 611–622. (10.1373/clinchem.2008.112797) 19246619

[bib4] Cakmak H & Taylor HS 2011 Implantation failure: molecular mechanisms and clinical treatment. Hum Reprod Update 17 242–253. (10.1093/humupd/dmq037) 20729534 PMC3039220

[bib5] Cavagna & Mantese 2003 Biomarkers of endometrial receptivity--a review. Placenta 24 (Supplement B) S39–S47. (10.1016/s0143-4004(03)00184-x) 14559029

[bib6] Coughlan C, Ledger W, Wang Q, et al. 2014 Recurrent implantation failure: definition and management. Reprod Biomed Online 28 14–38. (10.1016/j.rbmo.2013.08.011) 24269084

[bib7] De G 2019 Assisted reproductive technology: impact on society and need for surveillance. Best Pract Res Clin Endocrinol Metab 33 3–8. (10.1016/j.beem.2019.01.004) 30799230

[bib20] Diedrich K, Fauser BC, Devroey P, et al. 2007 The role of the endometrium and embryo in human implantation. Hum Reprod 13(Supp. 4) 365–377. (10.1093/humupd/dmm011) 17548368

[bib8] Elnaggar A, Farag AH, Gaber ME, et al. 2017 AlphaVBeta3 Integrin expression within uterine endometrium in unexplained infertility: a prospective cohort study. BMC Womens Health 17 90. (10.1186/s12905-017-0438-3) 28950833 PMC5615471

[bib9] Esteves SC, Humaidan P, Roque M, et al. 2019 Female infertility and assisted reproductive technology. Panminerva Med 61 1–2. (10.23736/s0031-0808.18.03553-x) 30674179

[bib10] Franasiak JM, Forman EJ, Hong KH, et al. 2014a Aneuploidy across individual chromosomes at the embryonic level in trophectoderm biopsies: changes with patient age and chromosome structure. J Assist Reprod Genet 31 1501–1509. (10.1007/s10815-014-0333-x) 25241130 PMC4389946

[bib11] Franasiak JM, Holoch KJ, Yuan L, et al. 2014b Prospective assessment of midsecretory endometrial leukemia inhibitor factor expression versus ανβ3 testing in women with unexplained infertility. Fertil Steril 101 1724–1731. (10.1016/j.fertnstert.2014.02.027) 24690239 PMC4101991

[bib12] Gamazon ER & Stranger BE 2015 The impact of human copy number variation on gene expression. Brief Funct Genomics 14 352–357. (10.1093/bfgp/elv017) 25922366 PMC4592354

[bib13] Gnainsky Y, Granot I, Aldo P, et al. 2015 Biopsy-induced inflammatory conditions improve endometrial receptivity: the mechanism of action. Reproduction 149 75–85. (10.1530/rep-14-0395) 25349438

[bib14] Granot I, Gnainsky Y & Dekel N 2012 Endometrial inflammation and effect on implantation improvement and pregnancy outcome. Reproduction 144 661–668. (10.1530/rep-12-0217) 23028125

[bib15] Henrichsen CN, Chaignat E & Reymond A 2009 Copy number variants, diseases and gene expression. Hum Mol Genet 18 R1–R8. (10.1093/hmg/ddp011) 19297395

[bib16] Ionita-Laza I, Rogers AJ, Lange C, et al. 2009 Genetic association analysis of copy-number variation (CNV) in human disease pathogenesis. Genomics 93 22–26. (10.1016/j.ygeno.2008.08.012) 18822366 PMC2631358

[bib17] Johnson GA, Burghardt RC, Bazer FW, et al. 2023 Integrins and their potential roles in mammalian pregnancy. J Anim Sci Biotechnol 14 115. (10.1186/s40104-023-00918-0) 37679778 PMC10486019

[bib18] Johnston-MacAnanny EB, Hartnett J, Engmann LL, et al. 2010 Chronic endometritis is a frequent finding in women with recurrent implantation failure after in vitro fertilization. Fertil Steril 93 437–441. (10.1016/j.fertnstert.2008.12.131) 19217098

[bib19] Lessey BA 1994 The use of integrins for the assessment of uterine receptivity. Fertil Steril 61 812–814. (10.1016/s0015-0282(16)56688-6) 8174714

[bib21] Lessey BA, Palomino WA, Apparao K, et al. 2006 Estrogen receptor-alpha (ER-alpha) and defects in uterine receptivity in women. Reprod Biol Endocrinol 4 (Supplement 1) S9. (10.1186/1477-7827-4-s1-s9) 17118173 PMC1679803

[bib22] Pös O, Radvanszky J, Buglyó G, et al. 2021 DNA copy number variation: main characteristics, evolutionary significance, and pathological aspects. Biomed J 44 548–559. (10.1016/j.bj.2021.02.003) 34649833 PMC8640565

[bib23] Seli E & Garcia-Velasco JA 2021 Editorial: assisted reproductive technology (ART) in a changing world. Curr Opin Obstet Gynecol 33 157–158. (10.1097/GCO.0000000000000699) 33896914

[bib24] Szamatowicz M 2016 Assisted reproductive technology in reproductive medicine - possibilities and limitations. Ginekol Pol 87 820–823. (10.5603/gp.2016.0095) 28098933

[bib25] Thornhill A, deDie-Smulders C, Geraedts J, et al. 2005 ESHRE PGD consortium 'best practice guidelines for clinical preimplantation genetic diagnosis (PGD) and preimplantation genetic screening (PGS). Hum Reprod 20 35–48. (10.1093/humrep/deh579) 15539444

[bib26] Ziegler SF & Buckner JH 2009 FOXP3 and the regulation of Treg/Th17 differentiation. Microbes Infect 11 594–598. (10.1016/j.micinf.2009.04.002) 19371792 PMC2728495

